# Facilitator and barrier perspectives on learning and implementing high-resolution anoscopy in Abuja, Nigeria: a qualitative study

**DOI:** 10.1186/s12885-025-15120-w

**Published:** 2025-11-14

**Authors:** Megan E. Mansfield, Connor R. Volpi, Chama John, Ruxton Adebiyi, Andrew Mitchell, Jumoke A. Aigoro, Yerima Jibrin Bawa, Kazeem E. Kolawole, Uchenna Ononaku, Paul Jibrin, Oluwole Olaomi, Francis Agbo, Abayomi Aka, Søren M. Bentzen, Stephen E. Goldstone, Patrick Dakum, Joel M. Palefsky, Cheryl Knott, Sylvia Adebajo, Rebecca G. Nowak

**Affiliations:** 1 Institute of Human Virology, University of Maryland, School of Medicine, Baltimore, MD USA; 2 Johns Hopkins Bloomberg School of Public Health, Baltimore, MD USA; 3 Institute of Human Virology Nigeria, Abuja, Nigeria; 4 National Hospital, Abuja, Nigeria; 5 National Agency for the Control of AIDS, Abuja, Nigeria; 6 International Center for Advocacy on Rights to Health, Abuja, Nigeria; 7 University of Maryland Greenebaum Comprehensive Cancer Center, Baltimore, MD USA; 8 Icahn School of Medicine at Mount Sinai, New York, NY USA; 9 University of California, San Francisco, San Francisco, CA USA; 10 University of Maryland, College Park, College Park, MD USA

**Keywords:** Anal cancer prevention, Implementation science, Africa

## Abstract

**Background:**

Early detection and treatment of anal precancer via high resolution anoscopy (HRA) is paramount to prevent anal cancer, particularly for populations at heightened risk like sexual minority men (SMM) living with HIV. Successful training and sustainability of cancer screening requires attention to local contexts, best captured by qualitative research. Using the Consolidated Framework for Implementation Research (CFIR), this study investigated factors that challenged or fostered learning and implementing HRA across a variety of stakeholder groups in Abuja, Nigeria.

**Methods:**

Using in-depth qualitative methodology, nineteen semi-structured interviews were conducted in September 2023 with stakeholders - patients who underwent HRA, HRA providers, and health system representatives in Nigeria. Thematic analysis, guided by CFIR, was employed to identify key themes related to the barriers and facilitators to practicing HRA as guided by the International Anal Neoplasia Society.

**Results:**

Eight themes were identified across three domains. Barriers included low knowledge and understanding of HRA, with participants explicitly noting the need for more research in low resource settings to garner local acceptance. Participants were concerned about financial costs for the clinic and the patients. Facilitators included organizational buy-in, SMM social networks, and a safe clinic environment to support HRA engagement. Facilitators important for sustainability included acceptance of the research evidence for HRA and recognition of the health benefits. Overall, participants from all stakeholder groups welcomed HRA as a new evidence-based intervention as part of HIV care services.

**Conclusions:**

Our study highlighted the need for localized research, cultural sensitivity, and resource allocation to improve the adoption of HRA in a Nigerian HIV care setting. Organizational buy-in, community engagement, and safe healthcare environments facilitated trust and patient engagement and would promote long term sustainability. Overall, the study provided perspectives from various stakeholders that strengthen clinical proficiency and sustainability of anal cancer screening in Nigeria.

**Supplementary Information:**

The online version contains supplementary material available at 10.1186/s12885-025-15120-w.

## Introduction

Anal cancer is the most prevalent human papillomavirus (HPV)-associated cancer among sexual minority men (SMM) living with HIV [1, 2], and its burden is expected to rise in low- and middle-income countries (LMICs) due to the high prevalence of HIV, oncogenic HPV, poor vaccine coverage, and improved survival [3–6]. With 30,146 new cases of anal cancer reported in 2020 [7, 8], many could be prevented with an expansion of anal cancer screening [9]. As demonstrated by the ANCHOR study [10], anal cancer can be prevented through the timely identification and treatment of high-grade squamous intraepithelial lesions (HSIL), the precursors to anal cancer, using high resolution anoscopy (HRA).

Anal cancer remains a significant but often overlooked public health concern in Nigeria, with an estimated crude incidence rate of 0.65 per 100,000 men and increasing age-specific rates observed particularly among those aged 45 years and older [11]. In 2020, over 100 new cases were reported annually among men aged 40–59, with corresponding mortality rates rising steeply with age [11]. Despite this growing burden, there are currently no official national recommendations or organized screening programs for anal cancer in Nigeria (e.g., usage of HRA) underscoring the urgent need for targeted prevention and early detection strategies.

Initiating anal cancer screening with HRA presents its own unique challenges because of the steep learning curve in correctly identifying anal HSIL [12, 13]. To help standardize anal HSIL detection, the International Anal Neoplasia Society (IANS) developed guidelines on clinic setup for HRA services, training and mentorship in HRA, practical competencies during HRA procedures, target practice volumes and performance metrics, and monitoring patient experiences [14]. However, the IANS guidelines were developed by experts from high-income settings who had clinical infrastructure, integrated pathology services, and accessible mentorship. Bringing anal cancer screening to Nigeria where there are unique LMIC context-specific challenges [9, 15] prompted an evaluation of whether the IANS guidelines and the screening could be readily integrated into HIV care services.

Qualitative methodology offers a nuanced and in-depth investigative approach for identifying context-specific barriers and facilitators for implementing a novel procedure [16], such as HRA-guided detection of HSIL, previously unavailable in Nigeria. Emerging qualitative research highlights the importance of obtaining three perspectives: patients, health providers, and health system for overall HRA sustainability [17–21]. Barriers highlighted by patients included lack of knowledge, limited access to screening, HIV or HPV-related stigma, and discomfort. Health provider barriers included gaps in knowledge and expertise, as well as challenges in communicating and building relationships with patients. System-level barriers encompassed societal stigma, limited healthcare capacity, and competency with new clinical procedures [18, 22–24]. Furthermore, barriers are resolved when a variety of bridges are built across stakeholder groups [18]. When providers adopt communication to convey knowledge to address patient-reported uncertainty, SMM express a willingness to engage in anal cancer screening [25]. However, most anal cancer screening research focuses on the patient [18–21] or provider perspective [26] but often lacks integration from all stakeholders. Sustaining anal cancer screening in Nigeria where the population who would benefit from the service is highly marginalized will require support from members of the targeted population, healthcare providers, and other health sector stakeholders [27, 28].

This study is part of a larger implementation research study, the Integrated Model for Prevention of Anal Cancer using screen and Treat for HSIL (IMPACT). One of the aims of the study is to identify barriers and facilitators to training and implementing HRA per the IANS guidelines in the local Nigerian setting [14]. The current study contributes to this aim by capturing the perspectives of patients, providers and health sector stakeholders in a setting naïve to HRA. Specifically, we were guided by the conceptual framework, Consolidated Framework for Implementation Research (CFIR) [29], because its consideration of the internal and external environment as well as the process and the complexity of the efficacious procedure (e.g., HRA) aligned with our goal to explore future sustainability in an LMIC setting.

## Methods

### Study setting

This research was conducted at the TRUST Clinic, a community-based research and care center established in 2012 in Abuja, Nigeria through a tripartite partnership between the International Center for Advocacy and Rights to Health (ICARH), the Institute of Human Virology Nigeria, and the University of Maryland Baltimore [30]. Through this collaboration, ICARH recruited a cohort of nearly 2,800 SMM using respondent-driven sampling, in which initial participants received coupons to recruit peers in their social networks. This peer-driven model fostered a sense of trust and confidentiality critical for engaging a highly stigmatized population [31]. The success of the TRUST cohort is in part attributed to the safety and freedom felt by participants who come to the clinic. Our prior qualitative research highlighted the TRUST clinic as a place where SMM could freely express themselves, a place they love to come to, and where it allowed them to readily engage with study and healthcare staff [32]. The clinic offers HIV and STI testing and treatment services and has become a trusted hub for healthcare and research among SMM in Abuja [33–35]. The broader study environment includes key health system actors and providers operating within this context. These stakeholders, providers, policymakers, and government officials are embedded in a health system where services are largely paid out of pocket, infrastructure is often limited, and training for specialized procedures such as HRA is scarce [36, 37].

A key component of the IMPACT study is the Implementation Science (IS) Team, which includes local stakeholders who have been involved with the study since its inception. IS Team members comprise patients, healthcare providers, researchers, and representatives from the government and non-governmental organizations. This multisectoral, community-engaged team provides a unique opportunity to explore both structural and interpersonal dynamics that shape the feasibility and acceptability of implementing HRA screening among Nigerian SMM. The primary objective of the IS Team is to identify and adapt elements of the IANS guidelines to the Nigerian context. To do this they meet regularly to review guideline components, discuss contextual barriers, and co-develop locally appropriate adaptations, such as modifying training benchmarks, adjusting language in patient-facing materials, and identifying culturally sensitive approaches to screening, to ensure the IANS guidelines are acceptable and feasible within the Nigerian healthcare and sociocultural context. As a result, of their knowledge of and engagement with the IANS guidelines, many members of the IS Team participated in the interviews conducted for the current study.

### Conceptual framework

CFIR was the theoretical framework used to guide this inquiry, allowing for a comprehensive examination of the contextual factors affecting implementation processes in an LMIC setting [38, 39]. CFIR categorizes conceptual elements from various theories and disciplines into 39 constructs, further organized into five key domains. These domains encompass intervention characteristics, outer setting, inner setting, characteristics of individuals, and implementation process [40], all of which interact to influence the implementation effectiveness of an evidence-based intervention [29]. Our qualitative interview guide prompted participants to provide nuanced perspectives on CFIR constructs in terms of their function as both barriers and facilitators for the implementation of HRA as per the IANS guidelines. We also asked participants what recommendations they had for overcoming barriers and leveraging facilitators. Specifically, they were prompted to consider their own experiences and opinions based on their area of expertise.

### Study design and sample

The semi-structured interviews (SSI) were conducted in September 2023 with participants from our three groups of interest – patients, health providers, and health system representatives (Table 1). Patients, aged 24–39, were purposefully sampled to include those who had good and moderate antiretroviral adherence, in order to capture variability in how participants follow health recommendations, including HIV medication adherence [41]. Patients were further stratified based on self-reported levels of pain experienced during the HRA procedure to ensure a range of procedural experiences. All participating patients had completed HRA at the TRUST Clinic within the prior 2–4 months. Most of these patients identified as bisexual or pansexual (75%) and the remaining identified as gay or homosexual (25%). Eligibility criteria for the SSI included being willing to attend an in-person interview at the TRUST Clinic and providing written informed consent. Participants received refreshments and approximately 8500 Naira (approximately $5.00 USD) to cover transportation costs.

**Table 1:** Participant demographic information (*n* = 19)

**Group**	**Participant**	**Affiliation**		
Healthcare Providers	Clinic Data Manager	TRUST		
	Clinic Nurse Provider	TRUST		
	Clinic Director	TRUST		
	Clinic Medical Doctor	TRUST		
Health System Representatives	Surgeon	National Hospital		
	Pathologist	National Hospital		
	Program Manager	International Center for Advocacy and Rights to Health		
	Executive Director	National Institute of Cancer Research and Treatment		
	Senior Program Manager	National Agency for the Control of AIDS		
	Senior Research Advisor	Institute of Human Virology Nigeria		
	Director	Representative of Private Sector		
**Patients**		**CFIR Criteria**	**Pain Score** ^*^	**ART Adherence**
	Patient 1	Good ART	4	≥ 95% of pills
	Patient 2	Good ART	1	≥ 95% of pills
	Patient 3	Moderate ART	5	50–95% of pills
	Patient 4	Moderate ART	2	50–95% of pills
	Patient 5	High pain	8	50–95% of pills
	Patient 6	High pain	8	≥ 95% of pills
	Patient 7	Low pain	1	50–95% of pills
	Patient 8	Low pain	1	≥ 95% of pills

*Pain score was self-reported on a scale from 0 (none) to 10 (highest) after undergoing high resolution anoscopy

*Abbreviation*: *ART* antiretroviral therapy

The health providers were clinicians directly involved in learning, conducting, and refining HRA procedures for detecting HSIL at the TRUST Clinic. Health system representatives included individuals holding roles at local hospitals, non-governmental organizations, and government agencies, all of whom had direct involvement with or oversight of the implementation of the HRA program, such as participating in strategic planning, training, or monitoring activities. Eligibility criteria for providers and health system representative groups included having direct knowledge of HRA procedures, being responsive to the interview invitation (via email or phone), and providing written informed consent to participate Interviews were conducted at a mutually agreed location (e.g., participant’s office), with the majority conducted at the TRUST Clinic.

Data collection continued until thematic saturation [42, 43] was reached within each stakeholder group, defined as the point at which no new themes or concepts emerged during interviews. The study team conducted ongoing, iterative reviews of the interviews throughout data collection to assess the emergence and repetition of themes specific to each group. Although formal member checking was not conducted, preliminary themes were reviewed and discussed by the broader team to support the credibility and contextual validity of the findings. Of note, potential stakeholders who were not responsive to multiple contact attempts were no longer pursued.

## Data collection

The publicly available CFIR Interview Tool [44] was adapted for the SSI guide (Appendix A). Two trained qualitative researchers conducted the interviews. The interviewers were both White, cisgender, and academically trained researchers from the U.S. who identify as sexual minorities and have prior experience conducting research with sexual and gender minority communities. The study team engaged in reflexivity throughout the study, recognizing how personal and professional standpoints shaped the design, conduct, and interpretation of the interviews [45, 46]. Prior to data collection and during analysis, team members discussed positionality and critically examined how their lived experiences may have influenced participant interactions and analytic decisions. The interview guide included directions on questions that were relevant to the different groups of participants and their areas of expertise to ensure their responses were relevant and appropriate (e.g., patients were not asked about provider training or clinical implementation procedures). This adaptive approach, grounded in reflexive practice [45, 46], supported the credibility and trustworthiness of the data by ensuring that participants were only asked questions aligned with their lived experience and contributions to HRA implementation. The interviews lasted 30–60 min and were audio recorded and transcribed verbatim by local members of our study team. As needed, a translator was present to support converting Hausa or Nigerian Pidgin into English. The translator was a bilingual member of the study team who had prior experience working with the TRUST Clinic population. To promote comfort and safety, particularly given the stigmatized identities of many participants, interviews were conducted in private settings, and participants were encouraged to use pseudonyms or initials instead of their real names during the interviews.

## Data analyses

We conducted a thematic analysis of the transcripts [47]. Thematic analysis is a flexible analytic procedure that focuses on the experiences of stakeholders in their own words [47, 48]. This approach allowed us to identify key themes related to both facilitators and barriers to implementing HRA per the IANS guidelines in Nigeria.

The analyses began with the researchers familiarizing themselves with the data through review of interviewer notes and transcripts. They then collaborated on an initial codebook with codes deductively specified based on CFIR. The initial codebook was pilot-tested on a subset of the transcripts (*n* = 5) using ATLAS.ti [49]. Piloting the codebook allowed the researchers to ensure they were using the codes consistently (i.e., calibration). Iterative discussions during the piloting process resulted in minor adjustments of the codebook to further facilitate consistent use of the codebook. These adjustments included identification of emergent codes and coming to agreement on coding practices (e.g., coding larger segments of text at the level of sentences or paragraphs to retain as much context as possible). Once calibrated, the researchers each independently coded the remaining 14 transcripts.

After coding was completed, the coded texts were grouped into themes and sub-themes and linked with CFIR constructs. The identified themes are presented with support from participant quotations. Throughout the coding and thematic analyses process, the coders engaged with local members of the research team to promote culturally sensitive interpretations. Reporting for this study was guided by the Consolidated Criteria for Reporting Qualitative Research (Table 2) [50].

**Table 2:** Responses to criteria for reporting qualitative studies checklist

**Domain 1: Research team and reflexivity**		
Personal Characteristics of Data Collectors		
1. Interviewers	Who conducted the interviews?	Two white American members of the IMPACT research team.
2. Credentials	What were their credentials?	Both interviewers had master’s level degrees related to social science research.
3. Occupation	What was their occupation at the time of the study?	One interviewer was a Senior Qualitative Analyst and a PhD student. The other interviewer was a PhD student.
4. Gender	Were they a man or woman?	One interviewer was a woman, and she conducted all internal team interviews. The other was a man, and he conducted all interviews with IMPACT participants. Both interviewers conducted interviews with external stakeholders.
5. Experience and training	What experience or training did they have?	One interviewer had 11 years of experience in conducting and analyzing qualitative research. She had a BA in LGBT studies and psychology, a master’s degree with a co-concentration in applied research methods and evaluation and applied social psychology. At the time of data collection, she was pursuing their PhD in applied social psychology and had been working with members of the research team for over a year. The other interviewer had over 7 years of experience in conducting and analyzing qualitative research. He has a BS in psychology, a master’s degree in public health with a focus in epidemiology. At the time of data collection, he was pursuing his PhD in Social & Behavioral Sciences and had been working with members of the research team for over a year.
Relationship with Participants		
6. Relationship established	Was a relationship established prior to study commencement.	For external stakeholders and IMPACT participants, the semi-structured interview conducted for this study was the first time the interviewer had met the interviewee. For internal team interviews, the interviewer had an established relationship with two of the four interviewees as they had worked together (virtually) for about one year.
7. Participant knowledge of the interviewer	What did the participants know about them?	Most participants knew the interviewers as members of the IMPACT study team and that their role was to conduct these semi-structured interviews. Some members of the internal team were professionally familiar with the interviewers.
8. Interviewer characteristics	What characteristics were reported about the interviewer?	None

**Domain 2: Study design**		
Theoretical Framework		
9. Methodological orientation and theory	What methodological orientation was stated to underpin the study?	Deductive coding based on CFIR framework
Participant Selection		
10. Sampling	How were participants selected?	Purposive and convenience sampling – we purposefully recruited stakeholders including patients, health providers, and health system representatives.
11. Method of approach	How were participants selected?	Purposive/convenience
12. Sample size	How many participants were in the study?	19
13. Non-participation	How many people refused to participate or dropped out?	2 external stakeholders were unresponsive to recruitment efforts
Setting		
14. Setting of data collection	Where was the data collected?	Most interviews were conducted in private spaces at the TRUST Clinic. Some external stakeholders were interviewed at their offices in other locations in Abuja
15. Presence of non-participants	Was anyone else present beside the participants and the researchers?	All IMPACT participant interviews included a translator who was also a member of the research team. The same translator was present for some of the external stakeholder interviews. A study coordinator was present for all internal team interviews.
16. Description of sample	What are the important characteristics of the sample?	Stakeholder group (i.e., patients, health provider, health system representative)
Data Collection		
17. Interview guide	Were questions, prompts, guides provided by the authors? Was it pilot tested?	An interview guide was developed based on the CFIR framework and refined based on findings from a study using the CFIR card game. It was not pilot tested.
18. Repeat interviews	Were repeat interviews carried out?	No
19. Audio/visual recording?	Did the research use audio or visual recordings to collect the data?	Audio recordings
20. Field notes	Were field notes made during and/or after the interviews?	Yes, the interviewers took notes during the interviews and completed a debrief online survey following each interview.
21. Duration	What was the duration of the interviews?	30–60 min
22. Data saturation	Was data saturation discussed?	Yes, the interviewers reviewed their notes and ensured saturation was reached before concluding data collection efforts.
23. Transcript returned	Were transcripts returned to participants for comments and/or correction?	No

**Domain 3: Analysis and findings**		
Data Analysis		
24. Number of data coders	How many data coders coded the data?	2
25. Description of the coding tree	Did authors provide a description of the coding tree?	Yes, codebook is provided.
26. Derivation of themes	Were themes identified in advance or derived from the data?	Themes were derived from the data based on inductive and deductive coding. \
27. Software	What software, if applicable, was used to manage the data?	Transcripts were created in Microsoft Word. Data was analyzed using ATLAS.ti
28 Participant checking	Did participants provide feedback on the findings?	Some participants were given the opportunity to review and provide feedback on the findings.
Reporting		
29. Quotations presented	Were participant quotations presented to illustrate the themes/findings? Was each quotation identified?	Yes, quotations were provided, and stakeholder group was acknowledged. But individual participants were not identified.
30. Data and finding consistent	Was there consistency between the data presented and the findings?	Yes
31. Clarity of major themes	Were major themes clearly presented in the findings?	Yes
32. Clarity of minor themes	Is there a description of diverse cases or discussion of minor themes?	Yes, inconsistencies or disagreements are described in the findings.

## Results

We completed 19 SSI, eight with patients enrolled in the IMPACT study (42%), four with providers at the TRUST clinic (21%), and seven with Nigeria health system representatives (37%). A primarily deductive thematic analysis of the codes revealed a total of eight themes across three domains (see Table 3). The themes reflect CFIR constructs in terms of both barriers and facilitators of implementing HRA based on the IANS guidelines.

**Table 3:** Themes and related CFIR constructs identified across three domains

*CFIR Construct*	*Theme*	*Summary*
**Domain 1: Barriers to HRA Implementation per the IANS Guidelines**		
Evidence Strength & Quality	More research on anal cancer screening is needed in Nigeria and other LMIC	Stakeholders acknowledged that research supporting the guidelines is primarily conducted with samples different from them.
Cost	Cost implications are a concern relating to necessary infrastructure to implement HRA and relating to the out-of-pocket expenses for patients	Costs of setting up clinics to conduct HRA and for patients to complete the screening.
**Domain 2: Facilitators of Implementing HRA per the IANS Guidelines**		
Self-Efficacy & Individual Stage of Change	Providers are supportive of and feel competent with the IANS guidelines	TRUST Clinic staff are willing and feel efficacious when implementing HRA.
Compatibility & Culture	The TRUST clinic is a safe space for patients	TRUST Clinic is a safe space for patients to receive potentially vulnerable services.
Networks & Communications	Social networks play an important role	Awareness and completion of HRA is increased through social relationships.
**Domain 3: Facilitators of Sustainability and Broader Implementation HRA**		
Evidence Strength & Quality (localized)	Existing research is well accepted	Despite limitations, stakeholders acknowledge the robustness and legitimacy of the guidelines.
Relative Advantage & Patient Needs and Resources	HRA serves the needs of patients	Stakeholders acknowledge that the guidelines are beneficial to health of TRUST patients and Nigerians more broadly.
Cosmopolitanism	Stakeholder engagement is crucial	Participants acknowledge the importance of engaging relevant people to promote acceptance and broader implementation of HRA as per the guidelines.

*Abbreviation*: *CFIR* Consolidated Framework for Implementation Research, *IANS* International Anal Neoplasia Society;, *HRA* high resolution anoscopy

### Domain 1: barriers to HRA implementation per the IANS guidelines

Overall, the two themes constituting barriers to implementing HRA screening using the IANS guidelines centered on feasibility, especially considering the potential expansion of HRA beyond the TRUST clinic.

#### Evidence strength and quality: more research on anal cancer screening is needed in Nigeria and other LMICs

Provider and health system stakeholders acknowledged that, even though they trusted the existing research that supports the IANS guidelines, this research is limited. Specifically, the guidelines were written primarily by Westerners and little to none of the research was conducted in Nigeria or other LMICs. One health system representative noted, “*all the evidence is there [in high income countries [but] …here [in Nigeria] you have to start from scratch to get that evidence*.” As a result, the feasibility and appropriateness of some of the aspects may require changes for improved training and ultimately implementation in Nigeria. For example, one provider suggested adjusting some of the milestones, such as detecting at least 50 HSILs during the first year of training: *“With time and then the longer we do it*,* 50 [HSIL detection] might begin to seem more realistic. But for now*,* given that it’s our first year we might not be hitting those figures.”* Additionally, another provider suggested adjusting the language used in some of the materials to be more sensitive to different cultural contexts, stating, *“there is this great diversity of tradition that we have…Nigeria is very*,* very large. So*,* some of the things you may need to tweak due to their beliefs of that area or that region…the language. For example*,* [for] some people the word “sex*,*” “anus*,*” or “cervix” may [or may not] be offensive to them*.”

#### Cost: costs implications are a concern relating to necessary infrastructure to implement HRA and relating to the out-of-pocket expenses for patients

Stakeholders from all three groups discussed barriers associated with costs. One provider stated that “*funding will be key…in terms of the actual training of these persons and letting them have a work tool.”* The tools refer to the costs of getting a facility equipped to complete HRA screenings as per the IANS guidelines as a notable barrier. Many commented that such expenses would require facilities to have funders, such as one health system representative who stated, “*I was asking myself particularly around things that need to be in place for the anoscopy. I’m asking myself who bears the costs of those things?*” But another health system representative disagreed stating that “*the necessary equipment [is] not too expensive…[for] smaller clinics*,* that should not be expensive.”*

However, both providers and health system representatives agreed that the mentorship component of HRA implementation is a notable challenge for training of Nigerian providers, noting “*it’s very expensive training one person”* (health system representative). To their knowledge, there were no mentors on the African continent, let alone in Nigeria. So, mentors must be flown in from abroad, which drastically increases the cost as compared to contexts wherein mentors are more localized.

Additionally, participants brought up the expenses associated with conducting HRA. Who pays for the screening, pathology, and treatment for any illnesses that are detected? Patients described difficulty getting to HRA appointments due to the cost of transportation. Although, as part of the larger study, their transportation was covered, many suspected that without that financial support most people would not be able to have access to HRA screening. One patient stated, “*what I think that can stop people can be cost of transportation because many people are living far away…the transportation cost that is what I see as a major setback*.” There was concern that, even if the screening was free, people would be hesitant to get screened given the potential implications for the cost of treatment based on the results of the screening. If anal cancer were detected, many would likely be unable to afford such treatments. One patient stated, “*the treatment – I know it’s a lot*,* it’s a very expensive thing. It is not an easy experience [receiving a cancer diagnosis] and there is no solution coming from anywhere.”* One health system representative further explained that “*most of the health services [in Nigeria] are paid out of pocket. So*,* if [patients] are going to bear the cost*,* you will see that it is going to be difficult.”*

### Domain 2: facilitators of implementing HRA per the IANS guidelines

Overall, the three themes in this domain demonstrate the importance of an awareness of the context in which HRA is being implemented. In the context of this study, with an emphasis on the SMM community in Nigeria, the reputation of the clinic and dynamics between staff and patients emerged as important facilitators.

#### Self-efficacy and individual stage of change: providers are supportive of and feel competent with the IANS guidelines

The providers were very supportive of and motivated to learn HRA and use the IANS guidelines. One provider described the clinic staff as being all *“on board. I believe everybody gets the message and everybody’s working to ensure that it’s been implemented the way it’s supposed [to be].”* Similarly, health system representatives expressed strong support for the implementation of anal cancer HRA screening with one representative stating “*I believe that it [anal cancer screening] enhances – help[s] the client to access care and get treated.”*

Providers and health system representatives also felt confident about their knowledge of the IANS guidelines, noting that their knowledge, understanding and confidence were growing over time. They especially emphasized the important role mentorship plays in supporting their increased knowledge and confidence. One provider described how another provider “*told me he was also not confident when he started*,* like we were just doing everything blindly…The [PROVIDER] is now very confident.”*

#### Compatibility and culture: the TRUST clinic is a safe space for patients

Participants from all groups emphasized the importance of the TRUST facility in terms of patient comfort. In Nigeria, SMM experience stigma and discrimination in community and societal contexts. As a result, SMM patients, the target population for this study, need to feel safe and accepted when attending appointments for a potentially stigmatizing procedure. One patient explained that “*If this procedure is taken to another hospital they may stigmatize or shame someone.”* So, many participants emphasized that a facility without this kind of acceptance and trust will not be able to implement anal cancer screening HRA well. For example, one health system representative explained that the “*TRUST Clinic is very much welcoming because you have the population over there. They understand what they are doing there. But if you take it to a normal general hospital*,* how would that affect their accessibility to the care? Their willingness to go? Will they be blackmailed? Will they be threatened?”*

#### Networks and communications: social networks play an important role

Patients discussed the important role of community social networks for increasing awareness of and accessing anal cancer screening. For example, one patient noted that “*if I have a friend that has it but does not want to undergo the screening*,* I will ask why and advise him to do it because it is an issue that can claim his life*.” Patients also described learning and sharing with others about HRA via WhatsApp groups and interpersonal relationships/conversations, emphasizing the importance of information dissemination with one patient stating, “*send information*,* more information. Enlighten us through WhatsApp groups*,* through the Facebook page and you know that is how we can do it one-on-one messages…a lot of us are in WhatsApp groups.*”

Social networks play a crucial role not only in sharing information but also in assisting patients with transportation to clinics for screening appointments. As members of a stigmatized group, one patient explained that traveling to the clinic in groups felt more comfortable: “*I’m even thinking bringing him here because sometimes they do fear in our community but they’re hiding. They don’t want to be out. So if you tell them*,* okay*,* go by yourself to the clinic*,* they will say*,* this is our first time*,* come lets go together*” with another stating that leveraging social connections can also increase attendance and help mitigate costs: “*I don’t have money to go with you if you can go alone. That is the same challenge I’m talking about. Bringing them here is the same challenge to me and you*.”

### Domain 3: facilitators of sustainability and broader implementation of HRA

The three themes in this domain demonstrate the utility and acceptance of anal cancer screening through HRA implementation among all stakeholder groups. Stakeholders respected the training and recognized the associated health benefits, which motivate patients to complete the procedures.

#### Evidence strength & quality: existing research is well accepted

Providers and health system representatives were confident and accepted the robustness and legitimacy of the IANS guidelines. One provider stated that they “*trust the IANS guidelines*.” Overall, they trusted the scientific rigor of the existing research. For example, one health system representative described reviewing presentations and journal articles about HRA and “*the findings have helped to know that these things can be practiced in other countries or in other areas. I feel it’s something that should be done and executed*.” The participants described confidence in the applicability of the IANS guidelines with limited adaptations.

#### Relative advantage & patient needs and resources: HRA serves the needs of patients

Participants from all three groups acknowledged that HRA is beneficial for the health of patients and Nigerians more broadly. One provider described that patients find benefits in completing the screening and are happy they did it, stating “*a lot of clients are happy that they just even performed the process. Even those who have some benign results*,* more of them are happy. So*,* I think it is satisfying that more services are being added.*” This was further supported by patients who noted that they “*will come back for [the screening] 100%.”* And a health system representative noted that the benefits of the HRA guidelines are that they are “*patient-centered*” and *“everything is to be tailored to a patient. So*,* it meets the needs of patients rather than the needs of the healthcare worker.”* Patients agreed, with one noting that “*80% of our community members are not protecting themselves during sex…and some of us*,* we don’t take our health serious. So*,* I think there is need for us to continue doing tests…I feel there is a need for us to do the test*,* we need it*.”

#### Cosmopolitanism: stakeholder engagement is crucial

Participants from all three groups acknowledged the importance of engaging relevant people for their support in promoting acceptance and broader implementation of HRA as per the IANS guidelines. They emphasized the need to partner not only with established community-based organizations that relate to and are trusted by SMM at risk for anal cancer, but also with key institutional stakeholders. As one participant explained, “*If you get the Ministry of Health*,* led by the Minister for Health*,* on board*,* then the rest is done*.” A health system representative simply stated, “*You have to be in the community as well. The linkage is the reason we have [access].”* Participants noted that this success has been observed in at the TRUST clinic due to the collaboration and engagement of the community-based organization ICARH. As one provider explained, “*we have gatekeepers*,* influencers*,* and key opinion leaders working with us*,* collaborating with over the years. So*,* these people were the persons who gave us access and gave us a channel to pass this information to the members within the community about something like this [screening.] Imagine we didn’t have those channels; it would be quite difficult to actually get the message out there and get people coming in for the procedure.”*

## Discussion

Using HRA to detect and treat HSIL is paramount to the prevention of anal cancer [1, 2] and with the availability of the IANS guidelines, its adoption is within reach in LMICs. Specifically, settings like Nigeria have the most to gain from this secondary prevention strategy, given the population of SMM living with HIV and the lack of clinical expertise [8]. Our analyses integrated perspectives from patients, HRA providers, and health system representatives to capture a comprehensive understanding of the facilitators and barriers to HRA implementation as per the IANS guidelines. Although presenting the data collectively allowed us to identify overarching themes, we acknowledge that each stakeholder group offered unique insights. Capturing local perspectives from various stakeholders on the facilitators and barriers to learning and implementing anal cancer screening is necessary for long term success and sustainability [51]. Nuanced differences provided from the different stakeholder groups offer valuable context for tailoring implementation strategies to address the distinct needs and priorities of each group. Specifically, we identified existing research limitations and concerns about the cost of implementing HRA as a barrier. General facilitators included provider and patient acceptability when the screening was based at a clinic where patients felt safe. Concerning overall sustainability, our analyses found evidence for acceptability across stakeholder groups for implementing HRA per the IANS guidelines suggesting a good foundation for building further buy-in from entities beyond the inner context of the TRUST Clinic.

The findings from this study shed light on the complex landscape surrounding the roll out of anal cancer screening in Nigeria, particularly within the context of the SMM community. Through an exploration of barriers, facilitators, and sustainability factors, several crucial themes emerged, offering relevant insights for public health practitioners, policymakers, and researchers alike. These insights can ultimately be used to inform sustainable implementation of HRA in Nigeria and serve as the foundation for building capacity in HRA in other LMICs.

The identified barriers underscore the need for contextual adaptation and resource allocation to ensure the successful integration of HRA into existing healthcare settings. The lack of localized research and cultural sensitivity within the existing guidelines present significant hurdles. These barriers were recently highlighted by Blair et al., who emphasized the need for data and strategies specific to LMICs [4]. Further, although there has been some research on HRA and anal cancer screening in Latin America, we are not aware of any study implementing HRA in Nigeria or any other country in Africa [52–55]. Efforts to bridge this gap should prioritize collaborative research endeavors that actively involve a variety of stakeholders (e.g., community members, researchers, clinicians, policy makers) and reflect the diverse sociocultural landscape of the country. Furthermore, mitigating the financial burdens associated with facility setup, mentorship costs, and patient out-of-pocket expenses is critical, as these barriers pose a substantial threat to the broader implementation and long-term sustainability of HRA programs in Nigeria and similar LMIC settings. Without strategic financial support, effort to scale up screening services may stall, particularly in resource-constrained contexts where most health services are paid for out-of-pocket. Strategies such as securing local or donor funding, integrating cancer screening into existing public health infrastructures, and implementing subsidized or free screening and treatment programs have been shown to enhance accessibility and feasibility in comparable LMIC settings [56–58]. These approaches can not only reduce financial strain on patients and providers but also build more sustainable and equitable screening programs. For the IANS guidelines specifically, the ability to obtain proper mentorship presented a significant hurdle. There are a limited numbers of HRA providers globally and they are primarily located in non-LMICs, making access for LMICs disproportionately costly [59].

The IANS guidelines [60] emphasize that effective implementation of HRA requires structured provider training, including supervised clinical practice and ongoing mentorship to ensure diagnostic accuracy and safety. Leveraging tele-mentorship platforms and identifying regional HRA providers to become future mentors can help address workforce limitations, reduce reliance on international experts, and enhance sustainability in resource-limited settings such as Nigeria [61, 62]. These strategies align with the preferences expressed by Nigerian providers in our study, who emphasized the importance of mentorship and context-specific adaptation of training benchmarks to match the local burden of disease and available infrastructure.

Conversely, facilitators identified in this study offer important leverage points for optimizing the implementation process. The supportive stance of HRA providers and other stakeholders, coupled with their increasing confidence in adhering to the IANS guidelines, is a testament to the importance of organizational buy-in and capacity development. This support is particularly needed for SMM who are hesitant to access care in more traditional settings [31]. The pursuit of supportive and safe spaces for Nigerian SMM in this cohort has been documented and reflects their intra-community solidarity despite stigma [32]. SMM relied on peers for various forms of support, while also valuing the services and sanctuary provided by the research site [32]. Creating a safe and inclusive environment within HIV care facilities is essential, particularly for marginalized populations, and is reliant on the pivotal role of healthcare providers in fostering trust and patient engagement. Moreover, harnessing community social networks not only enhances awareness but also fosters a sense of collective empowerment and support, bolstering HRA uptake. Community engagement has been reported to increase other types of cancer prevention in Nigeria with recent studies reporting that such engagements led to notable rises in cervical cancer screening, clinical breast examination, and HPV vaccination uptake [63]. In addition to community engagement, engagement of policy makers and national organizations (e.g., Ministry of Health, National Institute for Cancer Research and Treatment) will be crucial for ensuring the sustainability and equitable accessibility of HRA in Nigeria. Although participants acknowledged this reality, especially in terms of mitigating patient costs, more discussion and work needs to be completed on how to effectively engage these entities of the outer context.

Many efforts were taken to ensure methodological rigor, including verbatim transcription of interviews, independent coding with subsequent consensus-building, and the engagement of local team members. However, the limited number of coders may have introduced bias or overlooked certain nuances in the data analyses process, especially given that the coders were both non-Nigerians. Additionally, although saturation (i.e., consistent responding such that additional interviews were unlikely to uncover new information or themes) was reached, the findings of this study are specific to the TRUST clinic and may not generalize to other contexts in Nigeria or other LMICs. Furthermore, the absence of consistent demographic data such as age, gender identity, and educational attainment limits our ability to contextualize participants’ perspectives and examine how experiences with HRA implementation may vary across subgroups. This omission reflects the original design of the interviews, which prioritized confidentiality and participant comfort, particularly given the sensitivity of the population and topic. These limitations highlight the need for additional research to explore a broader range of perspectives and employ more diverse methodological approaches to enhance contextualized understandings of facilitators and barriers to implementation of anal cancer screening in other LMICs.

This study examined barriers and facilitators to the implementation of HRA as per the IANS guidelines in Nigeria by integrating multiple perspectives from patients, providers, and healthcare system representatives. The study stakeholders’ unwavering confidence in the IANS guidelines and the perceived benefits of HRA affirm the true potential to address critical health needs related to anal cancer within the Nigerian population. Sustaining this momentum requires ongoing collaboration with key stakeholders, including established organizations with existing ties to target populations. By fostering strategic partnerships and leveraging the infrastructure established through initiatives like the IMPACT study, the scalability and sustainability of HRA programs can be significantly enhanced.

## Conclusion

Successful implementation of HRA in Nigeria requires a multifaceted approach that addresses both structural barriers and leverages existing community facilitators. By prioritizing contextually relevant adaptations, fostering organizational support, and cultivating community engagement, stakeholders can work towards realizing the full potential of HRA in advancing public health outcomes and reducing the burden of preventable diseases. These insights can inform sustainable HRA implementation in Nigeria and provide a foundation for scaling anal cancer screening in other LMICs.

## Supplementary Information

Supplementary Material 1.

## Data Availability

The data analyzed for the current study are not publicly available due to their sensitivity but are available from the corresponding author on reasonable request. Data collection materials, including the interview guide, are included in the supplemental materials.

## Abbreviations

HRA: High resolution anoscopy
SMM: Sexual minority men
CFIR: Consolidated Framework for Implementation Research
IANS: International Anal Neoplasia Society
CDC: Centers for Disease Control and Prevention
HIV: Human immunodeficiency viruses
LMICs: Low- and middle-income countries
HSIL: High-grade squamous intraepithelial lesion
IMPACT: Integrated model for prevention of anal cancer using screen and treat for HSIL
ICARH: International Center for Advocacy and Rights to Health
SSI: Semi-structured interviews
