# Supplementary material for: Facilitator and barrier perspectives on learning and implementing high-resolution anoscopy in Abuja, Nigeria: a qualitative study
Source: BMC Cancer. 2025 Nov 14;25:1766. doi: 10.1186/s12885-025-15120-w (PMC12619308; doi:10.1186/s12885-025-15120-w)
Supplement: Supplementary file 1 — Supplementary Material 1. [file 12885_2025_15120_MOESM1_ESM.docx]

**Appendix A. Semi-Structured Interview Guide**

INTERVIEW INTRODUCTION

Thank you for agreeing to complete this interview today! We really appreciate you taking the time. I just want to take a few moments to clarify our roles here and the purpose of our conversation today. My name is [name], and I will be the one primarily asking you to guide the conversation and asking you questions. This is [name], and they will be ensuring our conversation gets captured through the audio recording and also taking notes, just in case something goes wrong with the audio recording. They will also help if our conversation requires clarifications.

We have not started recording yet but once we formally start the interview we will. The purpose of the recording is to capture what you share with us today accurately. This way we do not have to write down everything and can instead focus on what you say. As a reminder everything you share today is confidential and we will never share any information that could potentially identify you. **Are you still okay with us doing the audio recording?**

Our conversation should take about one hour. The purpose of this interview is to take a deep dive into your thoughts on potential challenges we might encounter when implementing the IANS training guidelines. So, I’m interested in hearing about your thoughts and opinions and there are no right or wrong answers.

If at any point, I say anything you do not understand please feel free to ask for clarification. And, if there is anything you are uncomfortable answering just let me know and we can skip that question.

There may be times during the interview when I ask you to expand or explain your answer further – this is to ensure I fully understand what you are sharing. There may be other times when I ask that we move onto the next question so that we get through all the questions in the time allotted.

**Do you have any questions before we get started?**

**START RECORDING**

**Okay, today is [date] and it is [time]. I’m with [participant ID] for their interview.**

INTERIVEW QUESTIONS

Thanks again for speaking with me today. You may recall during the focus group discussion a few weeks ago you discussed components of the Consolidated Framework for Implementation Research – or CFIR for short. You played a card game and looked at aspects of five different domains – intervention and individual characteristics, inner and outer setting, and process. **Does this sound familiar?**

*Provide more details if the participant struggles to recall.*

Great! We will be discussing those barrier cards again.

**Intervention Characteristics**

We will start with intervention characteristics, which broadly covers aspects of the IANS training guidelines themselves.

1. **What do you think of the IANS training guidelines?** [source]
2. **Is the evidence or proof behind the IANS training guidelines sufficient to get you on board? Why or why not?** [evidence strength & quality]

In a healthcare setting there are many influential stakeholders like influential and well-respected clinicians, administrators, clinic staff, patients and clients. And in educational settings there are also teachers or educators.

1. **So, thinking about these people, what do you believe they would think about the IANS training guidelines?** [source]

*Remember to tailor this based on who you are speaking to (do not ask about participants’ role)*

- 1. Administrators
  2. Leaders (clinicians, teachers, educators)
  3. Staff
  4. Patients/clients

1. **For these people, do you think the evidence behind the IANS training guidelines is sufficient? Why or why not?** [evidence strength & quality]

*Remember to tailor this based on who you are speaking to (do not ask about participants’ role)*

- 1. Administrators
  2. Leaders (clinicians, teachers, educators)
  3. Staff
  4. Patients/clients

1. **What kinds of changes or alterations do you think we will need to make to the IANS training guidelines so it will work effectively in Nigeria?** [adaptability]
   1. **Do you think you will be able to contribute to these changes? Why or why not?**
2. **Are there components that you think should not be altered? Which ones and why?** [adaptability]
3. **Who do you think should decide whether changes are needed to the training guidelines so that it works well?** [adaptability]

*Prompt (if needed): What is the best process for deciding this?*

***For PATIENTS, skip to next domain.***

Now I want to ask you a bit about the complexity of the IANS training guidelines.

1. **In your opinion, how complicated are the training guidelines?** [complexity]
   1. Duration
   2. Scope
   3. Intricacy
   4. Number of steps involved
   5. Is the training clearly different from standard practices?
2. **What supports, such as online resources, marketing materials, or a toolkit, are available to help you with the training guidelines?** [design quality & packaging]
   1. **How do you access these materials?**
3. **How do the available materials affect the training guidelines in Nigeria?** [design quality & packaging]
4. **What costs will be incurred to implement the training guidelines?** [cost]

*Prompt (if needed): Money, resources*

1. **What costs were considered when deciding to implement the IANS training guidelines?** [cost]

**Outer Setting**

Great! We finished the first domain, now we are moving onto outer settings.

1. **How well do you think the IANS training guidelines will meet the needs of the patients served by the TRUST clinic?** [patient needs & resources]
   1. **In what ways will the IANS training guidelines meet their needs?**

*Prompts (if needed): Improved access to services?*

*Reduced pain?*

*Reduced negative perceptions of screening and treatment?*

*Improved understanding of cancer risk?*

1. **How do you think patients served by the TRUST clinic will respond to the IANS training guidelines?** [patient needs & resources]
2. **What challenges will the training guidelines create for the patients at the TRUST clinic?** [patient needs & resources]

***For PATIENTS, skip to next domain.***

Okay, I want to shift gears a bit now and I want you to think about your professional network, particularly those outside of your immediate work setting such as family members, friends, and community or religious leaders.

**External**

**4a. What kind of information exchange do you have those people in your professional network who are outside of your immediate work setting that might relate to the IANS training guidelines?** [cosmopolitanism] – external

*Prompts (if needed): Professional networks, local or national conferences, trainings.*

**Internal**

**4b. To what extent does the TRUST clinic encourage you to network with co-workers outside of your immediate work setting?** [cosmopolitanism] – internal

*Prompts (if needed): Are you able to attend local or national conferences? Other venues?*

**Inner Setting**

Let’s talk about the next domain now – inner setting. As a reminder, this is focused on the characteristics of the place where the IANS guidelines would be implemented, like the TRUST clinic for example.

1. **What kinds of infrastructure changes would be needed to accommodate the IANS training guidelines?** [structural characteristics]

*Prompts (if needed): Change in scope of practice?*

*Changes in formal policies?*

*Changes in information systems or electronic record systems?*

1. **Based on your understanding, what would the process be for making these changes?** [structural characteristics]
   1. **What kinds of approvals would be needed?**
   2. **Who would need to be involved?**
2. **How receptive is the TRUST clinic to implementing the IANS training guidelines?** [implementation climate]
3. **What kinds of incentives or rewards would be needed for people to be motivated to accommodate the IANS guidelines?** [implementation climate/organizational incentives and rewards]

*Prompts (if needed): Facility staff, healthcare providers, leadership*

- 1. **What would be the process for making those possible?**
  2. **Who would need to be involved?**

***For PATIENTS, skip to next domain.***

So, thinking about the resources needed to implement the IANS training guidelines…

1. **Do you think there are sufficient resources to implement and administer the training guidelines?** [available resources]

**IF YES**

**What resources are you counting on?**

**IF NO**

**What are the resources you would need that would not be available?**

1. **What resources do you think people implementing the IANS guidelines will need if they have questions about the training guidelines or its implementation?** [available resources]

*Prompts (if needed): People to whom they can ask questions?*

*Online or paper resources?*

**6a. How easily accessible are these resources?**

**Characteristics of Individuals**

Thank you for sharing all of these thoughts and ideas with me, we only have two domains left!

1. **Do you think the IANS training guidelines will be effective in Nigeria?** [knowledge & beliefs]

*Prompts (if needed): Why or why not?*

1. **How do you feel about the training guidelines being used in Nigeria?** [knowledge & beliefs]

*Prompts (if needed): Do you have any feelings of anticipation? Stress? Enthusiasm?*

*Why?*

1. **[internal stakeholders]**
   1. **How confident are you that you will be able to successfully implement the training guidelines?** [self-efficacy]
   2. **How confident do you think your colleagues feel about implementing the training guidelines?** [self-efficacy]

**[external stakeholders]**

- 1. **How confident are you that you will be able to use the training guidelines?** [self-efficacy]
  2. **How confident do you think your peers feel about using the training guidelines?** [self-efficacy]

**[patients]**

- 1. **How confident are you in your ability to consistently follow the new training guidelines for your condition or treatment** [self-efficacy]
  2. **How confident are you that you can effectively incorporate the recently provided training guidelines into your routine?** [self-efficacy]

**[external/internal stakeholders]**

1. **How prepared are you to use the IANS training guidelines?** [individual stage of change]
   1. **Do you have knowledge of the key aspects of the training guidelines?** [precontemplation]
   2. **Do you like the training guidelines?** [contemplation]
   3. **Do you use the training regularly?** [action]
   4. **Have you integrated the training guidelines into your routines?** [maintenance]

**Process**

Thank you so much for powering through all of these questions.

1. **Other than formal implementation leaders, are there people in your organization who are likely to champion (i.e., go above and beyond what might be expected) the IANS training guidelines?** [champions]
   1. **What position do these champions have in your organization?**
   2. **How do you think they will help with implementation?**
   3. **Can you describe people’s perception of these champions?**

*Prompts (if needed): To what extent do you respect the opinion of these champions?*

1. **Will someone (or a team) outside your organization be helping with implementing the training guidelines?** [external change agents]

**IF YES**

- 1. **How will they help? And to what extent will their help be useful?**

1. **What steps have been taken to encourage individuals to commit to using the IANS training guidelines?** [key stakeholders]

*Prompts (if needed): How will you approach them?*

*How frequently and how will you communicate with them?*

1. **What is your communication or education strategy for getting the word out about the IANS training guidelines?** [key stakeholders]

*Prompts (if needed): What process do you plan to use to communicate?*

1. **Who are the key individuals to get on board with the IANS training guidelines?** [key stakeholders]

*Prompts (if needed): To encourage individuals to use the training guidelines?*

*To help with implementation?*

**Facilitators**

Now, I want to shift gears for a moment and ask you what might help the implementation of the IANS training guidelines instead of the challenges. So, thinking about the barriers you mentioned (*describe some of them to remind the participant)*

1. **What do you think are some ways these barriers could be addressed to help facilitate implementation of the guidelines.**
2. **Are there other facilitators – or things that might help implement the guidelines – that come to mind?**

*Prompts (if needed): Are there facilitators for specific barriers that come to mind?*

**Closing**

Thank you for sharing all of that information with me! I know it was a lot of question and some of them were difficult to answer, but your efforts will certainty help us adapt the guidelines so that they are appropriate and well used in Nigeria.

We have reached the end of my prepared questions.

1. **Is there anything else you think we should have talked about that I did not bring up?**
2. **Do you have anything else you would like to share?**

Great! Let’s go ahead and stop the recording. Then we can wrap-up.

**STOP RECORDING**

INTERIVEW DEBRIEF

Great, so before I let you go, we just have a couple housekeeping items.

First, I just want to let you know that – as a member of the implementation team you may be invited back to do another focus group in a few months.

Now, before we provide you with your compensation, **do you have any questions?**

*Provide participant with compensation funds.*
